# Effects of immersive training on motor competence: a systematic review and meta-analysis

**DOI:** 10.1038/s41598-026-42962-6

**Published:** 2026-03-26

**Authors:** Yuyang Ji, Bin Wang, Qingqiong Yang

**Affiliations:** School of Physical Education, Yunnan Normal University, Kunming, 650500 China

**Keywords:** Augmented reality, Virtual reality, Motor competence, Immersive training, Meta-analysis, Motor skills, Health care, Medical research, Neuroscience

## Abstract

Augmented reality (AR) and virtual reality (VR) immersive training have gained attention for enhancing motor skills and rehabilitation, yet evidence on their effects in specific motor competence domains remains inconsistent due to outcome heterogeneity. This systematic review and meta-analysis evaluated the impact of immersive AR and/or VR training on domain-specific motor competence (stability and functional mobility; object control and visuomotor skills) in healthy individuals and athletes. We searched Web of Science, PubMed, Cochrane Library, Scopus, CNKI, and EBSCO from inception to October 2025 for randomized controlled trials and controlled intervention studies comparing immersive AR and/or VR training with conventional training or no-intervention controls. The review followed PRISMA 2020 guidelines and was prospectively registered in PROSPERO (CRD420251238825). Eligible studies involved healthy or athletic participants reporting motor performance outcomes, classified into domain-specific constructs: stability and functional mobility (e.g., locomotor skills/mobility, postural stability) and object control and visuomotor skills (e.g., complex sport-specific skills, fundamental visuomotor abilities). These domains were further grouped by skill type, predominantly closed skills (predictable environments) versus open skills (unpredictable, dynamic environments). A random-effects model was used to compute standardized mean differences (SMD; Hedges’ g), with heterogeneity assessed via I^2^ statistics and robustness via leave-one-out sensitivity analysis. Eighteen studies contributing 20 independent effect sizes were included (total participants = 678; intervention group = 341; control group = 337). Domain-specific analyses revealed moderate beneficial effects: stability and functional mobility (8 studies) SMD = 0.68 (locomotor skills/mobility subgroup SMD = 0.71; postural stability subgroup SMD = 0.62); object control and visuomotor skills (11 studies) SMD = 0.72 (complex sport-specific skills subgroup SMD = 1.15; fundamental visuomotor abilities subgroup SMD = 0.39). Sensitivity analyses confirmed robustness, with no single study substantially altering pooled estimates. Immersive AR/VR training demonstrates moderate beneficial effects in targeted motor competence domains, particularly stability/functional mobility and object control/visuomotor skills. Current evidence suggests these technologies serve as effective alternatives or complementary adjuncts to conventional training rather than superior replacements. However, substantial heterogeneity in some domains (e.g., I^2^ = 79% in locomotor skills/mobility) limits interpretability and warrants caution in generalization. Future research should prioritize standardized outcome measures and address methodological inconsistencies. This systematic review and meta-analysis was conducted in accordance with the Preferred Reporting Items for Systematic Reviews and Meta-Analyses (PRISMA) 2020 guidelines. The study protocol was prospectively registered in the International Prospective Register of Systematic Reviews (PROSPERO) under registration number CRD420251238825.

**Supplementary Information:**

The online version contains supplementary material available at 10.1038/s41598-026-42962-6.

The rapid advancement of immersive technologies, including AR and VR, has extended their applications from entertainment to sports science and rehabilitation medicine^1,2^. VR constructs fully simulated environments through head-mounted displays (HMDs) or immersive systems, generating a strong sense of presence^3^; In contrast, AR overlays digital elements onto the real world, enabling real-time interaction between physical and virtual objects^4,5^. These technologies provide multimodal sensory feedback (visual, auditory, and haptic) and incorporate gamified design and real-time performance monitoring, thereby potentially enhancing user engagement and neuroplasticity^6^, offering a comparable alternative to conventional methods particularly where space, equipment access, or safety concerns limit traditional practice^7,8^. Traditional motor training is often constrained by limitations in space, equipment, and feedback latency, making it difficult to practice complex or high-risk scenarios^7^. By comparison, AR and VR enable safe, repeatable simulation of domain-specific motor tasks—such as postural stability on unstable surfaces or visuomotor skills involving object interception in dynamic environments—with individualized adjustments^8^. These technologies align with established motor control theories, including perception-action coupling (where sensory feedback optimizes movement patterns) and ecological dynamics (adaptation under task constraints)^9–11^. In this review, motor competence is operationally defined as domain-specific constructs, comprising (1) stability and functional mobility (e.g., locomotor skills/mobility, postural stability assessed via the Berg Balance Scale or Timed Up and Go test) and (2) object control and visuomotor skills (e.g., fundamental visuomotor abilities such as reaction time/accuracy, and complex sport-specific skills such as agility or ball control)^12–15^. This classification draws on developmental motor frameworks (e.g., Gallahue’s hierarchy of fundamental motor skills) and distinguishes closed skills (predictable, self-paced tasks, such as static balance) from open skills (unpredictable, externally paced tasks, such as interception actions)^12^. The rationale for pooling AR and VR lies in their shared immersive mechanisms, which induce neuroplastic changes in the cerebellum and frontoparietal networks, thereby enhancing cross-domain sensorimotor integration^11,16^. Although individual trials demonstrate potential benefits, evidence for AR/VR effects remains inconsistent, influenced by outcome heterogeneity and small sample sizes. For instance, preliminary reviews report variable improvements in balance (SMD ≈ 0.6–0.8) and visuomotor performance (SMD ≈ 0.4–1.2), with substantial heterogeneity in many domains (I^2^ > 50%), precluding broad conclusions^4,17–19^. Prior meta-analyses have not addressed construct validity through domain-specific pooling. This systematic review and meta-analysis therefore evaluates the effects of immersive AR/VR training on the aforementioned motor competence domains in healthy individuals and athletes. Recent systematic reviews targeting athletes or healthy adults further highlight differential VR effects on open skills (e.g., visuomotor coordination) versus closed skills (e.g., balance), reinforcing the need for domain-specific analyses using random-effects meta-analysis and sensitivity testing for robustness^20,21^.

## Materials and methods

### Search strategy

The literature search adhered to the PRISMA 2020 statement^22^. Two independent reviewers (A and B) performed the systematic searches, with discrepancies resolved by a third reviewer (C). The following electronic databases were searched from inception to October 15, 2025: CNKI, PubMed, Web of Science, EBSCO, Cochrane Library, and Scopus. The search was initially conducted in April 2025 and updated in October 2025 to ensure completeness and currency of the evidence.

Search terms combined controlled vocabulary (e.g., MeSH terms) with free-text keywords^23^. Chinese and English search strings underwent bilingual cross-validation to ensure terminological consistency. No language or geographic restrictions were applied. Grey literature (e.g., conference proceedings, dissertations, registered trials) was excluded from the primary analysis, but reference lists were manually screened to identify any potentially eligible studies missed by electronic searches.

Chinese search string: (运动员OR健康人群OR临床人群) AND (虚拟现实OR增强现实OR头戴式显示器OR沉浸式OR体感游戏) AND (运动OR锻炼OR训练OR体育OR身体活动OR康复OR有氧运动) AND (平衡OR力量OR运动表现).

English search string: (athletes OR healthy individuals OR clinical populations) AND (virtual reality OR augmented reality OR head-mounted displays OR immersive experiences OR motion-sensing games) AND (physical activity OR exercise OR training OR sports OR rehabilitation OR aerobic exercise) AND (balance OR strength OR athletic performance).

### Inclusion and exclusion criteria

This review adopted the PICOS framework (Population, Intervention, Comparison, Outcomes, Study Design) to define eligibility criteria^24^.

Inclusion Criteria: (1) Population: Healthy individuals (including athletes, general adults, and students) or clinical populations (older adults). No restrictions on age or sex. Participants were required to be capable of performing basic physical movements and to have no history of severe cognitive impairment or psychiatric disorders. (2) Intervention: Any form of immersive VR or AR training that incorporated a physical motor component, compared with conventional training or no training. Eligible interventions included, but were not limited to, AR treadmill walking, AR proprioceptive exercise, VR sports games, VR cognitive-motor dual-task training, and VR aerobic-resistance training. Delivery devices could include head-mounted displays (HMDs), motion-sensing equipment (e.g., Kinect, HTC Vive), or immersive display systems. (3) Comparison: Conventional motor training (e.g., standard rehabilitation or physical exercise) or no-intervention/wait-list control groups. (4) Outcomes: Measures of motor competence, classified into two domain-specific constructs: Stability and Functional Mobility: Including postural stability (e.g., Berg Balance Scale [BBS], single-leg stance time) and locomotor skills/mobility (e.g., Timed Up and Go Test [TUG], 6-Minute Walk Test [6MWT], gait parameters, functional jumping). Object Control and Visuomotor Skills: Including complex sport-specific skills (e.g., agility tests, ball control, Hexagon Agility Test, reaction time/accuracy) and fundamental visuomotor abilities (e.g., hand-eye coordination, visual tracking, simple/choice reaction time [RT]). Outcomes were further categorized by motor skill type based on developmental motor frameworks^12,13^; Closed skills: Tasks performed in predictable, self-paced environments (e.g., static balance or gait locomotion), primarily corresponding to the stability and functional mobility domain; Open skills: Tasks performed in unpredictable, externally paced environments (e.g., reaction time tests or ball interception), primarily corresponding to the object control and visuomotor skills domain; Mixed skills: Protocols combining elements of both (e.g., standing long jump + Hexagon Agility Test in^25^, classified into the predominant domain based on primary components. 5) Study Design: Randomized controlled trials (RCTs) or quasi-experimental studies reporting sufficient data (pre- and post-intervention means, standard deviations [SD], and sample sizes) to calculate effect sizes.

Exclusion Criteria: (1) Intervention mismatch: Studies in which VR/AR interventions lacked a physical motor component or were used solely for assessment/measurement rather than training; (2) Study design mismatch: Studies without a control group, single-arm pre-post designs, reviews, case reports, or commentaries; (3) Population mismatch: Studies involving participants with severe neurological conditions substantially impairing cognitive or motor function; (4) Studies with inaccessible full texts or missing key data (means, SDs, sample sizes) that could not be obtained after contacting authors; (5) Publication type: Reviews, editorials, conference abstracts, book chapters, or other grey literature.

### Study selection and data extraction

All retrieved records were imported into Zotero reference management software for duplicate removal. Two independent reviewers screened titles and abstracts against the predefined inclusion and exclusion criteria. Full texts of potentially eligible studies were then retrieved and reviewed for final inclusion. Disagreements were resolved through discussion or arbitration by a third reviewer. A standardized data extraction form was used to collect the following information from included studies: 1): General study characteristics (e.g., first author, year of publication). 2): Participant characteristics (e.g., age, sample size per group), intervention details (e.g., type of AR/VR technology, intervention duration and frequency), and comparator details. 3): Baseline and post-intervention data for the outcomes of interest, including means, standard deviations (SDs), and sample sizes for both intervention and control groups to enable effect size calculation.

### Quality assessment

Two independent reviewers assessed the risk of bias in included studies using the Cochrane Risk of Bias tool in Review Manager 5.4 software (provided by the Cochrane Collaboration). Assessments were cross-verified, with discrepancies resolved by consensus or consultation with a third reviewer. Each study was evaluated across the following domains: randomization process, allocation concealment, blinding of participants and personnel, blinding of outcome assessment, incomplete outcome data, selective reporting, and other potential sources of bias. Domains were rated as low risk, high risk, or unclear risk. Studies judged to have an overall high risk of bias were considered for exclusion in sensitivity analyses to ensure the robustness of meta-analysis findings.

### Statistical analysis

All statistical analyses were performed using Review Manager 5.4 software (RevMan; Cochrane Collaboration). For continuous outcomes, the treatment effect was expressed as the standardized mean difference (SMD; Hedges’ g) with 95% confidence intervals (95% CI). Given anticipated clinical, methodological, and statistical heterogeneity across studies (e.g., differences in participant characteristics, intervention protocols, and outcome measures), a random-effects model was pre-specified for pooling effect sizes to better account for true between-study variation. This meta-analysis included 18 studies, but some reported multiple independent motor competence outcomes (e.g., different test items), yielding 20 independent effect sizes in total. These were allocated to domain-specific random-effects models: stability and functional mobility (7 studies, 8 effect sizes) and object control and visuomotor skills (11 studies, 12 effect sizes). All effect sizes were treated as independent, as no highly correlated outcomes (e.g., *r* ≥ 0.5) appeared within the same subgroup. Heterogeneity was assessed using Cochrane’s Q statistic (Chi^2^ test), with *P* ≤ 0.10 indicating statistical heterogeneity. The I^2^ statistic quantified the proportion of total variation attributable to between-study differences (range 0%–100%). Following Cochrane Handbook guidance^24^, I^2^ was interpreted approximately as follows: 0%–40% low (acceptable), 40%–60% moderate, 60%–75% substantial, and 75%–100% considerable. These thresholds are approximate and should be interpreted in context with effect direction, magnitude, evidence strength, and number of studies. When substantial heterogeneity was observed (e.g., I^2^ > 50% or Chi^2^ *P* ≤ 0.10), pre-specified subgroup analyses by domain (stability and functional mobility vs. object control and visuomotor skills) were conducted to explore potential sources. Subgroup analyses were exploratory in nature, aimed at identifying possible effect modifiers, and therefore no adjustment for multiple comparisons (e.g., Bonferroni correction) was applied. Interpretation of subgroup differences was cautious, integrating effect size magnitude, heterogeneity, and clinical relevance rather than relying solely on statistical significance. Publication bias was assessed when ≥ 10 studies contributed to an analysis: funnel plots were visually inspected for asymmetry, and Egger’s linear regression test was used to statistically evaluate asymmetry. For analyses with < 10 studies, only visual inspection of funnel plots was performed, with cautious interpretation due to limited statistical power. To examine the robustness of pooled results, sensitivity analyses were conducted by sequentially excluding individual studies (leave-one-out method) and recalculating pooled effect sizes. Changes in point estimates, direction, or 95% CI crossing zero were evaluated. Results are presented in forest plots to illustrate the influence of each study on the overall estimate. All statistical tests were two-sided, with *P* < 0.05 considered statistically significant.

## Results

### Literature inclusion

**Fig. 1:**
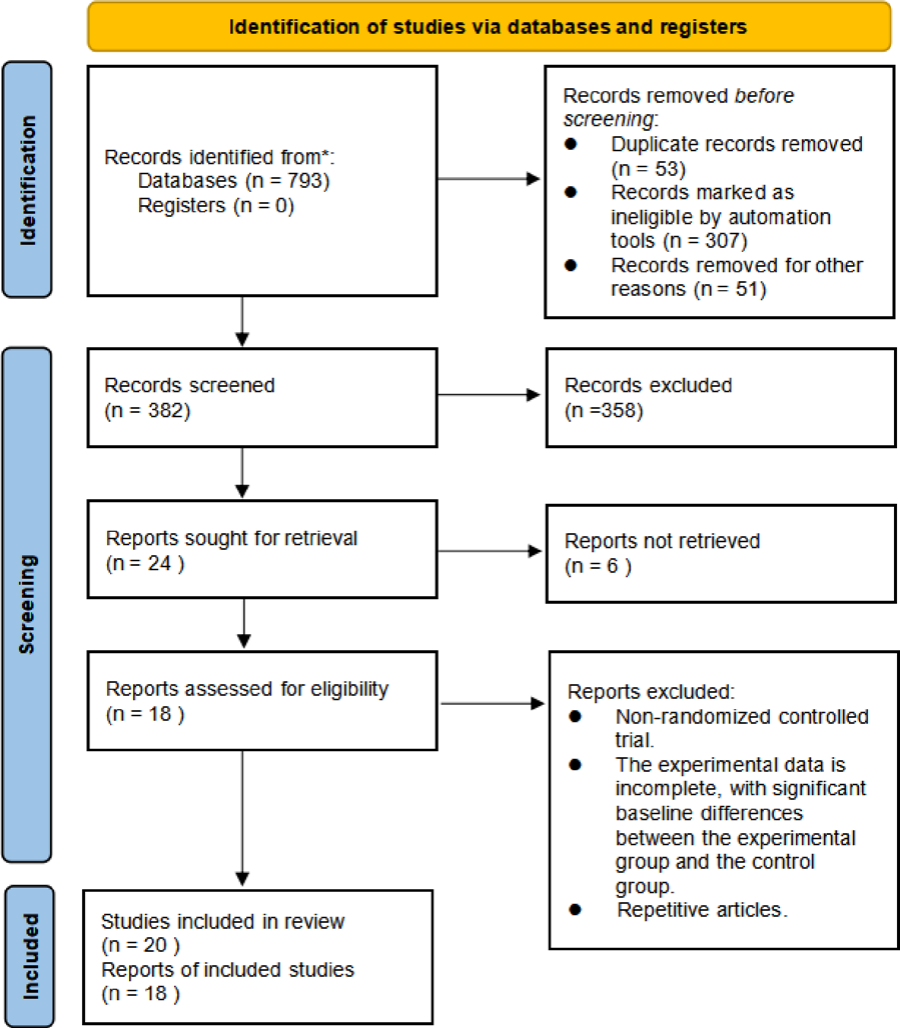
PRISMA flow diagram of the study selection process.

Figure 1 presents the PRISMA flow diagram illustrating the study selection process. A total of 793 records were identified from the searched databases (Web of Science, PubMed, Cochrane Library, Scopus, CNKI, and EBSCO), with no additional records from registers. After removal of 53 duplicates, 307 records marked ineligible by automation tools, and 51 records excluded for other reasons, 382 unique records remained for title and abstract screening. Of these, 358 were excluded based on the predefined inclusion and exclusion criteria (e.g., interventions lacking a physical motor component, absence of a control group, non-original research, or ineligible populations). Full-text assessment was conducted on the remaining 24 reports, of which 6 were excluded (5 due to inability to retrieve the full text and 1 due to missing key statistical data that could not be obtained after author contact). Ultimately, 18 studies (reported in 18 reports) met all inclusion criteria and were included in the qualitative synthesis and quantitative meta-analysis, contributing 20 independent effect sizes (8 in the stability and functional mobility domain and 12 in the object control and visuomotor skills domain).

### Characteristics of included studies

This meta-analysis included 18 studies (involving 20 independent analyses), encompassing a total of approximately 678 participants. Key characteristics of the included studies are summarized in Table 1. The studies were published between 2013 and 2025, with sample sizes ranging from 7 to 34 participants per study (total: intervention group = 341; control group = 337). Participants spanned diverse age groups, including adolescent athletes, adult exercisers, and older adults, with mean ages ranging from 9.3 ± 0.8 years to 75.6 ± 5.6 years. Interventions comprised AR-based training, VR-based training, conventional exercise, or no-intervention controls. Devices used included HTC Vive, Oculus Quest, Nintendo Wii Fit, AR treadmills, and other immersive systems. In terms of technology type, 13 studies employed VR, while 5 utilized AR. The included studies assessed motor performance using four main categories of outcome measures, corresponding to different dimensions of motor skills: (1) Locomotor skills and mobility: Evaluation of basic displacement and functional movement, primarily corresponding to closed skills. (2) Postural stability: Assessment of the ability to maintain body center of mass under static or dynamic conditions to prevent falls, primarily corresponding to closed skills. (3) Complex motor skills: Evaluation of performance in open, unpredictable environments involving sport-specific or task-oriented skills, corresponding to open skills. (4) Fundamental visuomotor abilities: Assessment of coordination and speed between visual information processing and motor execution, serving as the foundation for open skills. These outcome categories align with the domain-specific constructs defined in the Methods section (stability and functional mobility; object control and visuomotor skills) and reflect the classification of closed skills (predictable, self-paced) versus open skills (unpredictable, externally paced).

**Table 1:** Characteristics of the included studies.

Study	Population (Mean Age ± SD)	Population Type	Intervention Type & Device / Immersion Level	Intervention Protocol (Content / Dose / Total Sessions)	Adherence / Completion Rate	Comparison Group (Description / Intensity)	Task Type	Outcome Domain & Measure
Huang et al.^26^]	IG: *N* = 30, Age = 71.9 ± 5.9 / CG: *N* = 30, Age = 72.1 ± 6.2	Healthy seniors living in the community	AR-based / AR interactive treadmill (JOHNSON 8.1T) / Medium (screen+video)	Content: AR treadmill walking training; Dose: 60 min/session (40 min walking), 3x/week, 12 weeks (total ~ 36 sessions)	NR	Conventional treadmill walking (without AR) / Matched duration & frequency	Closed Skill	Locomotor Skills & Mobility (TUG, Gait)
Chitjamnogchai et al.^27^	IG: *N* = 26, Age = 69.2 ± 4.8 / CG: *N* = 27, Age = 71.2 ± 5.9	Elderly sarcopenic patients living in the community	VR-based / MiniPC + dance mat + Polar OH1 / Medium (screen-based)	Content: Home-based VR aerobic + resistance training; Dose: 60 min/session (5 + 30+20 + 5 min), 3x/week, 12 weeks (total ~ 36 sessions)	NR	Education only (knowledge about exercise) + usual activities / None	Closed Skill	Locomotor Skills & Mobility (6MWT)
Yoo et al.,^15^	IG: *N* = 10, Age = 72.9 ± 3.4 / CG: *N* = 11, Age = 75.6 ± 5.6	Older women with sufficient cognitive ability to participate (MMSE ≥ 24)	AR-based / Head-mounted display (i-visor FX601) + webcam / Medium (screen+model feedback)	Content: Augmented reality-based Otago exercise; Dose: 60 min/session, 3x/week, 12 weeks (total ~ 36 sessions)	NR	Otago exercise without AR / Matched duration & frequency	Closed Skill	Locomotor Skills (Velocity) & Postural Stability (BBS)
Park et al.^28^	IG: *N* = 12, Age = 66.5 ± 8.1 / CG: *N* = 12, Age = 65.2 ± 7.9	Healthy seniors living in the community	VR-based / Nintendo Wii Fit (balance board) / Medium (screen+avatar feedback)	Content: Virtual reality exercise (tennis, baseball, bowling); Dose: 40 min/session, 3x/week, 6 weeks (total ~ 18 sessions)	NR	Stable surface exercise (similar movements) / Matched duration & frequency	Closed Skill	Postural Stability (Sway Length)
Nekar et al.^29^	IG: *N* = 12, Age = 24.8 ± 3.1 / CG: *N* = 12, Age = 23.9 ± 2.6	Healthy adult men who engage in recreational sports activities	AR-based / UNICARE-82 AR device / Medium (screen+real-time feedback)	Content: Squat exercise with AR feedback; Dose: 4 sets × 30 reps, 3x/week, 4 weeks (total ~ 12 sessions)	100% (no drop-out)	Control (no feedback, blank wall) / Matched squat exercise	Closed Skill	Postural Stability (Static Balance)
Lee et al.^30^	IG: *N* = 19, Age = 22.7 ± 2.9 / CG: *N* = 20, Age = 21.8 ± 1.4	Healthy young adults	AR-based / AR device (type NR) / Medium (screen+visual feedback)	Content: AR-based proprioceptive exercise on Swiss Ball; Dose: 4 weeks (exact dose NR) / NR	NR	Physical therapist-led proprioceptive exercise / Matched exercise program	Closed Skill	Postural Stability (Dynamic Balance)
Michalski et al.^31^	IG: *N* = 29, Age = 22.1 ± 4.3 / CG: *N* = 28, Age = 21.5 ± 2.8	Healthy young adults (novice table tennis players)	VR-based / HTC Vive HMD, Two controllers / High immersion	Content: Playing competitive table tennis matches against AI opponent in VR (Eleven: Table Tennis VR). Dose: 30 min/session, ~2x/week, 7 sessions (total 3.5 h). Total: ~7 sessions.	NR	Description: No-training control group. Intensity: None.	Open Skill	Complex Sport Skills (Score)
Bedir & Erhan.^32^	IG: *N* = 12, Age = 21.1 ± 2.3 / CG: *N* = 11, Age = 23.8 ± 5.2	Target-based sports athletes (Curling, Bowling, Archery)	VR-based / VR glasses with 3D video / High immersion.	Content (VRBI): Progressive muscle relaxation + watching 3D performance video in VR + imagery + real shot practice. Dose: ~2 h/session, 3x/week, 4 weeks. Total: ~12 sessions.	NR	Description: Control group (watched fun videos about the sport before shooting practice). Intensity: Low.	Open Skill	Complex Sport Skills (Shooting)
Stefan.^33^	IG: *N* = 8, Age = 17–19 / CG: *N* = 8, Age = 17–19	High school students	VR-based; Oculus Quest 2; High (HMD full immersion)	Content: Exergames (Reakt, Thrill of Fight, etc.); Dose: 40 min/session, 2x/week, 12 weeks (total 24 sessions)	NR	No specific training program / None	Open Skill	Complex Sport Skills (Catching)
Günar & Bavlı.^34^	IG: *N* = 9, Age = 15.2 ± 0.8 / CG: *N* = 8, Age = 15.2 ± 0.8	Adolescent students (no prior basketball training)	VR-based / Oculus Quest 2, Oculus Gym Class Basketball VR / High immersion	Content: VR basketball training (ball control, dribbling, shooting). Dose: 15 min VR + 45 min court/session, 2x/week, 8 weeks. Total: ~16 sessions (VR).	17/22 completed (77.3%)	Description: Court-only training group (same 45-min on-court training, no VR). Intensity: Matched duration for on-court training.	Open Skill	Complex Sport Skills (Dribbling)
Benizar et al.^25^	IG: *N* = 30, Age = 18.0 ± 1.1 / CG: *N* = 30, Age = 18.8 ± 1.2	Young female athletes (Pencak Silat & Karate)	AR-based / AR application (device unspecified) / Medium immersion (assumed, as AR overlays animation on real world)	Content: Warm-up + AR-guided physical fitness (plank, squat jump, etc.) and technical exercises (punching/kicking) using virtual avatar + cool-down. Dose: 50 min AR training/session, 2x/week, 11 weeks. Total: ~22 sessions.	All 60 completed (100%)	Description: Traditional training (usual activities like running, push-ups, direct technical coaching without AR). Intensity: Matched session frequency and duration.	Mixed	Locomotor Skills (Jump - Closed) / Complex Sport Skills (Agility - Open)
Casella et al.^35^	IG: *N* = 12, Age = 24.5 ± 2.5 / CG: *N* = 12, Age = 24.5 ± 2.5	Healthy young college students	VR-based, Meta Quest 2 / High immersion (HMD)	Content: Reaction wall task (Rezzil Player); Dose: 30 min/session, 1x/week, 5 weeks (total 5 sessions)	NR	No training, only repeated testing / None	Open Skill	Fundamental Visuomotor (Sprint RT)
Lachowicz et al.^36^	IG: *N* = 32, Age = 24.0 ± 3.9 / CG: *N* = 34, Age = 24.0 ± 3.9	Amateur e-sports athletes	VR-based, Beat Saber (Valve Index VR) / High immersion (HMD)	Content: Beat Saber rhythm game; Dose: 15 min/session, daily, 8 consecutive days (total 8 sessions)	Dropout: 21.22%	No intervention / None	Open Skill	Fundamental Visuomotor (Hand-Eye RT)
Amprasi et al.^37^	IG: *N* = 16, Age = 9.3 ± 0.8 / CG: *N* = 16, Age = 9.3 ± 0.8	Children (female volleyball players)	VR-based, PlayStation4 VR / High immersion (HMD)	Content: “Climbing Wall” game; Dose: 24 min/session, 2x/week, 6 weeks (total 12 sessions)	NR	Typical court training / Matched duration, frequency	Open Skill	Fundamental Visuomotor (Whole-body RT)
Gürbüz & Taş.^38^	IG: *N* = 6, Age = 20.8 ± 0.8 / CG: *N* = 6, Age = 22.9 ± 0.9	Football goalkeepers	VR-based; Rezzil Player^®^ app (device NR); Immersion level NR	Content: Goalkeeper-specific drills (Reaction Wall, G.K Drills); Dose: 30 min/session, 2x/week, 6 weeks (total 12 sessions)	NR	Regular team training (unspecified content/intensity)	Open Skill	Fundamental Visuomotor (Go/No-Go RT)
Rutkowski et al.^39^	IG: *N* = 15, Age = 22.0 ± 1.8 / CG: *N* = 16, Age = 22.0 ± 1.8	University physiotherapy students	VR-based; HTC Vive Pro; High (HMD full immersion)	Content: Beat Saber rhythm game; Dose: 15 min/session, 1x/day, 5 days (total 5 sessions)	31 out of 32 completed (96.9%)	No intervention (wait-list) / None	Open Skill	Fundamental Visuomotor (Hand-Eye)
Witte et al.^40^	IG: *N* = 15, Age = 17.4 ± 3.5 / CG: *N* = 12, Age = 17.4 ± 3.5	Young karate athlete	VR-based; HTC Vive Pro Eye; High (HMD full immersion)	Content: Responding to virtual opponent attacks; Dose: 10 min VR + 80 min conventional/session, twice weekly for 5 weeks per phase (total 10 VR sessions over 10 weeks)	NR	Conventional karate training (90 min/session, matched duration/frequency) / Unspecified intensity	Open Skill	Fundamental Visuomotor (RT)
Petri et al.^41^	IG: *N* = 8, Age = 15.0 ± 1.7 / CG: *N* = 7, Age = 15.1 ± 1.2	Young karate athlete	VR-based; Oculus Rift DK2; High (HMD full immersion)	Content: Responding to virtual opponent attacks; Dose: 10–15 min/session, unspecified weekly frequency, 6 weeks (total 10 sessions)	NR	Conventional karate training (unspecified intensity)	Open Skill	Fundamental Visuomotor (RT)

Data are presented as Mean ± Standard Deviation (SD) or number of participants (N). For studies reporting only total sample characteristics, demographic values are applied to both groups unless otherwise specified.Abbreviations: NR = Not Reported; AR, Augmented Reality; BBS, Berg Balance Scale; CG, Control Group; HMD, Head-Mounted Display; IG, Intervention Group; RT, Reaction Time; TUG, Timed Up and Go Test; VR, Virtual Reality; 6MWT, 6-Minute Walk Test.Units: min, minutes; wks, weeks.

Task Type Definitions: Closed Skill: Motor tasks performed in a predictable, self-paced environment (e.g., walking, static balance). Corresponds to the Stability and Functional Mobility domain.Open Skill: Motor tasks requiring adaptation to an unpredictable, externally-paced environment (e.g., visual reaction, ball games). Corresponds to the Object Control and Visuomotor Skills domain.Mixed: Indicates protocols (e.g., Benizar, M. et al.) combining elements of both Closed (Standing Long Jump) and Open (Hexagon Agility) skills.

### Quality assessment of included studies

**Fig. 2:**
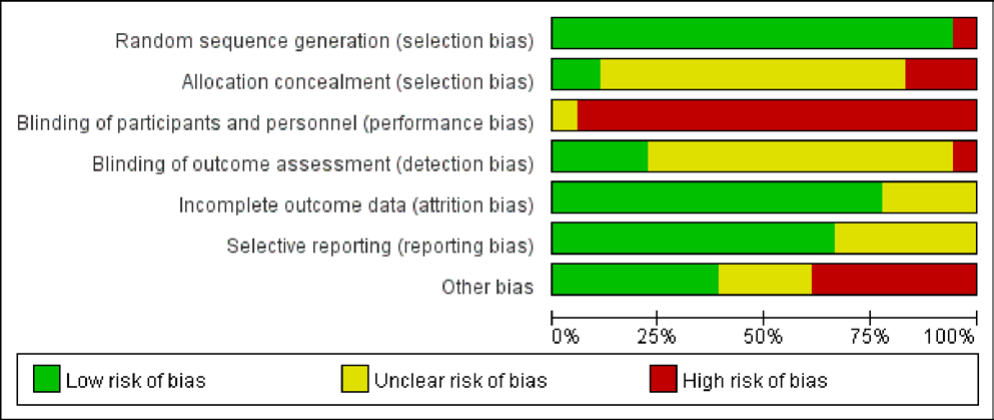
Methodological quality assessment diagram.

**Fig. 3:**
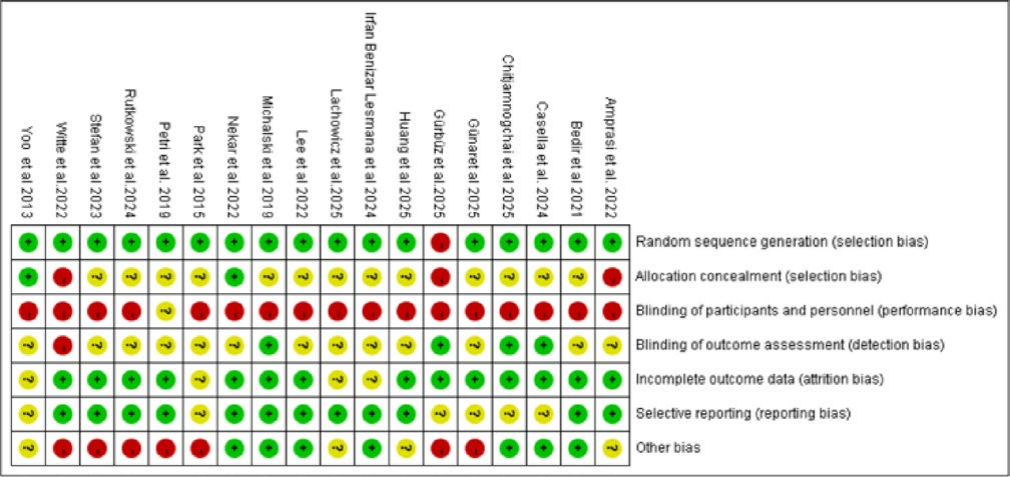
Methodological quality assessment item proportion.

Risk of bias was assessed in the 18 included studies using the Cochrane tool in Review Manager 5.4. All studies had complete data for evaluation. Figure 2 shows the risk of bias summary for each study across the seven domains. Figure 3 presents the percentage of studies rated as low risk (green), unclear risk (yellow), or high risk (red) for each domain.

### Sensitivity analysis

To verify the robustness of the main findings and evaluate the influence of individual studies on the pooled effect sizes, leave-one-out sensitivity analyses were performed by sequentially excluding each of the 20 effect sizes and recalculating the pooled standardized mean difference (SMD) with 95% confidence intervals.In response to reviewer suggestions, an additional sensitivity analysis was conducted by excluding studies that used no-intervention/wait-list controls^27,31^. After exclusion, the pooled SMD for stability and functional mobility changed from 0.68 to 0.72, and for object control and visuomotor skills from 0.72 to 0.65. In both cases, the 95% CI remained excluding zero, heterogeneity changes were minimal (I^2^ fluctuations < 10%), and the direction and statistical significance of effects were unchanged. These results indicate that the main conclusions are insensitive to the type of control group.

**Fig. 4:**
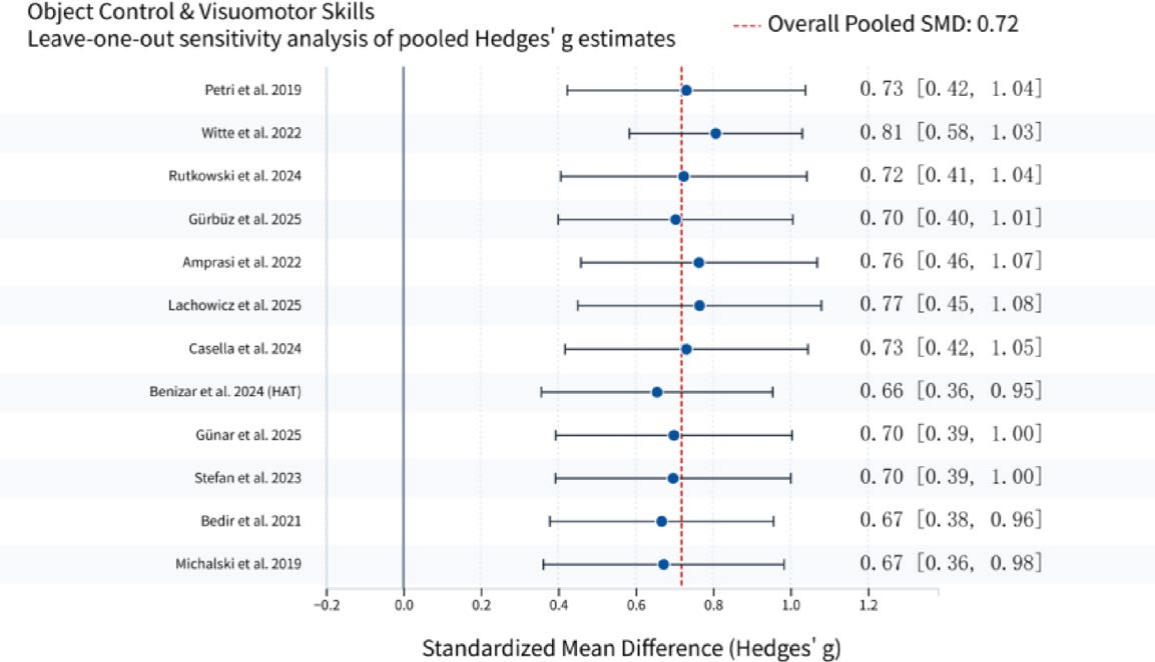
Sensitivity Analysis of Object Control and Visuomotor Skills.

Sensitivity analyses demonstrated high robustness for the pooled results in the object control and visuomotor skills domain. After excluding any single study, the recalculated Hedges’ g ranged from 0.66 to 0.81. No individual study dominated the overall estimate, and all recalculated effect sizes remained statistically significant (*p* < 0.05). Results are presented in Fig. 4.

**Fig. 5:**
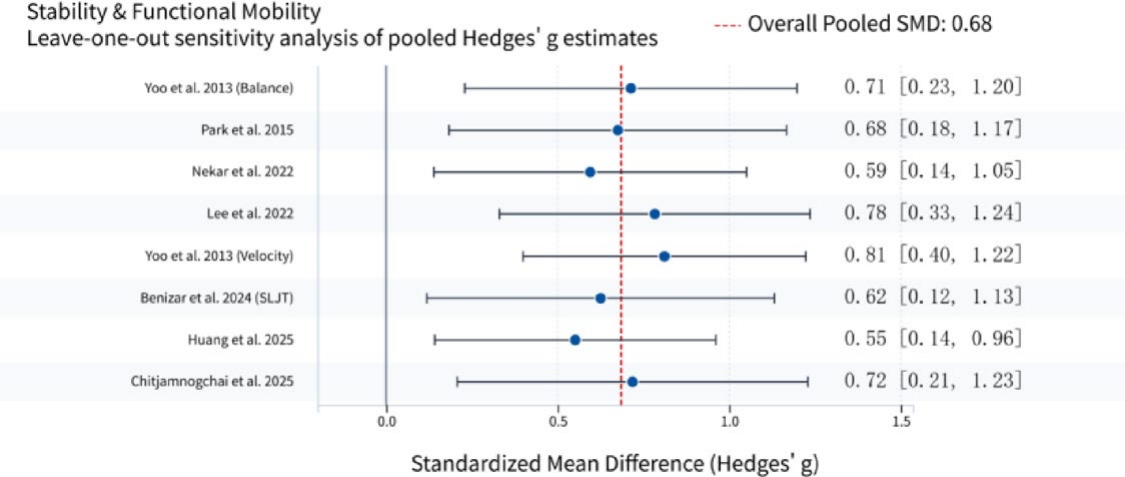
Sensitivity Analysis of Stability and Functional Mobility.

Sensitivity analyses for the stability and functional mobility domain similarly confirmed the robustness of the results. After sequential exclusion of individual studies, the pooled SMD varied slightly: exclusion of (Huang et al., 2025) resulted in a modest decrease to 0.55, while exclusion of^15^, velocity outcome led to an increase to 0.81. However, in all cases, the 95% confidence intervals excluded zero, indicating that the overall direction and statistical significance of the intervention effect were not unduly influenced by any single study. Results are presented in Fig. 5.

### Meta-analysis of object control and visuomotor skills

**Fig. 6:**
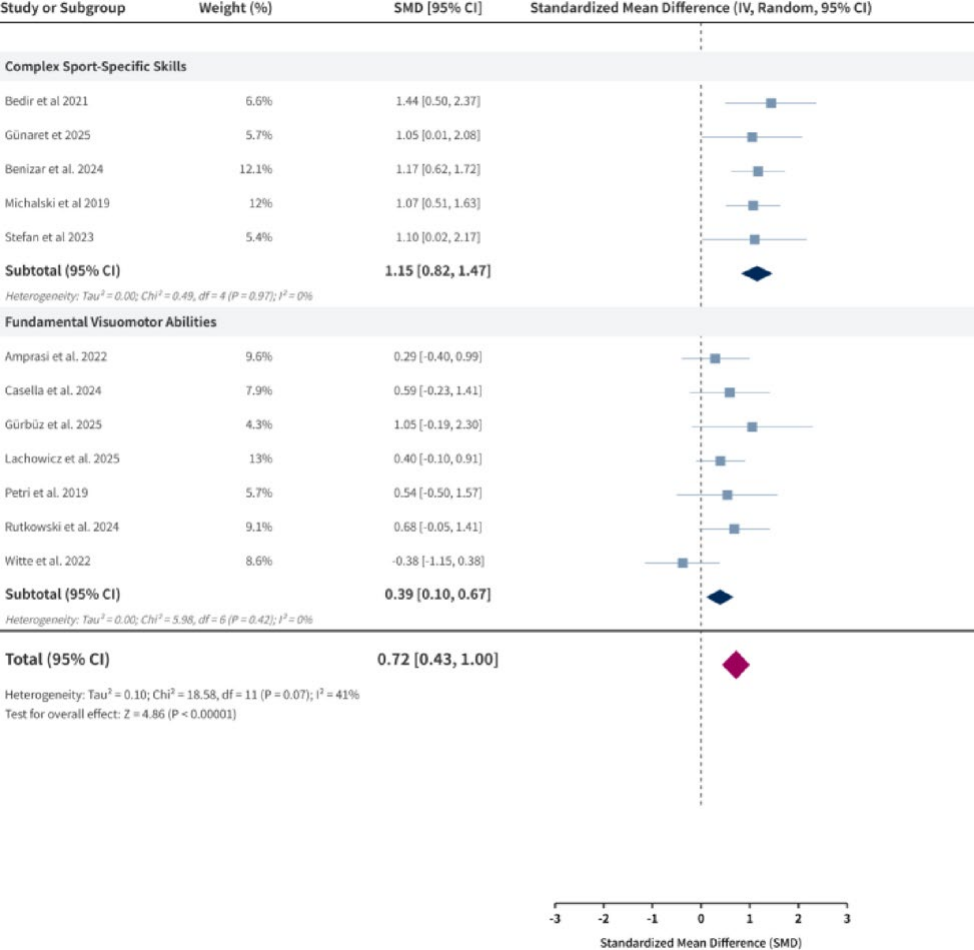
Object Control and Visuomotor Skills.

Subgroup analyses within the object control and visuomotor skills domain revealed significant differences in intervention effects across skill types. The overall pooled effect size was moderate (SMD = 0.72, 95% CI: 0.43, 1.00). When stratified by skill complexity (Fig. 6), complex sport-specific skills showed a large intervention effect (SMD = 1.15, 95% CI: 0.82, 1.47), with no heterogeneity within the subgroup (I^2^ = 0%). However, given the small sample sizes in the contributing studies (typically 7–34 participants per group), this large effect estimate may be inflated by small-study effects and should be interpreted cautiously as preliminary pending confirmation in larger, well-powered trials. In contrast, fundamental visuomotor abilities demonstrated a smaller improvement (SMD = 0.39, 95% CI: 0.10, 0.67), also with no within-subgroup heterogeneity (I^2^ = 0%). The markedly different effect sizes between these subgroups suggest that immersive AR/VR training is more effective for enhancing complex motor skills than for fundamental visuomotor abilities, though this pattern requires cautious interpretation in light of sample size limitations and the need for further validation.

### Meta-analysis results for stability and functional mobility

**Fig. 7:**
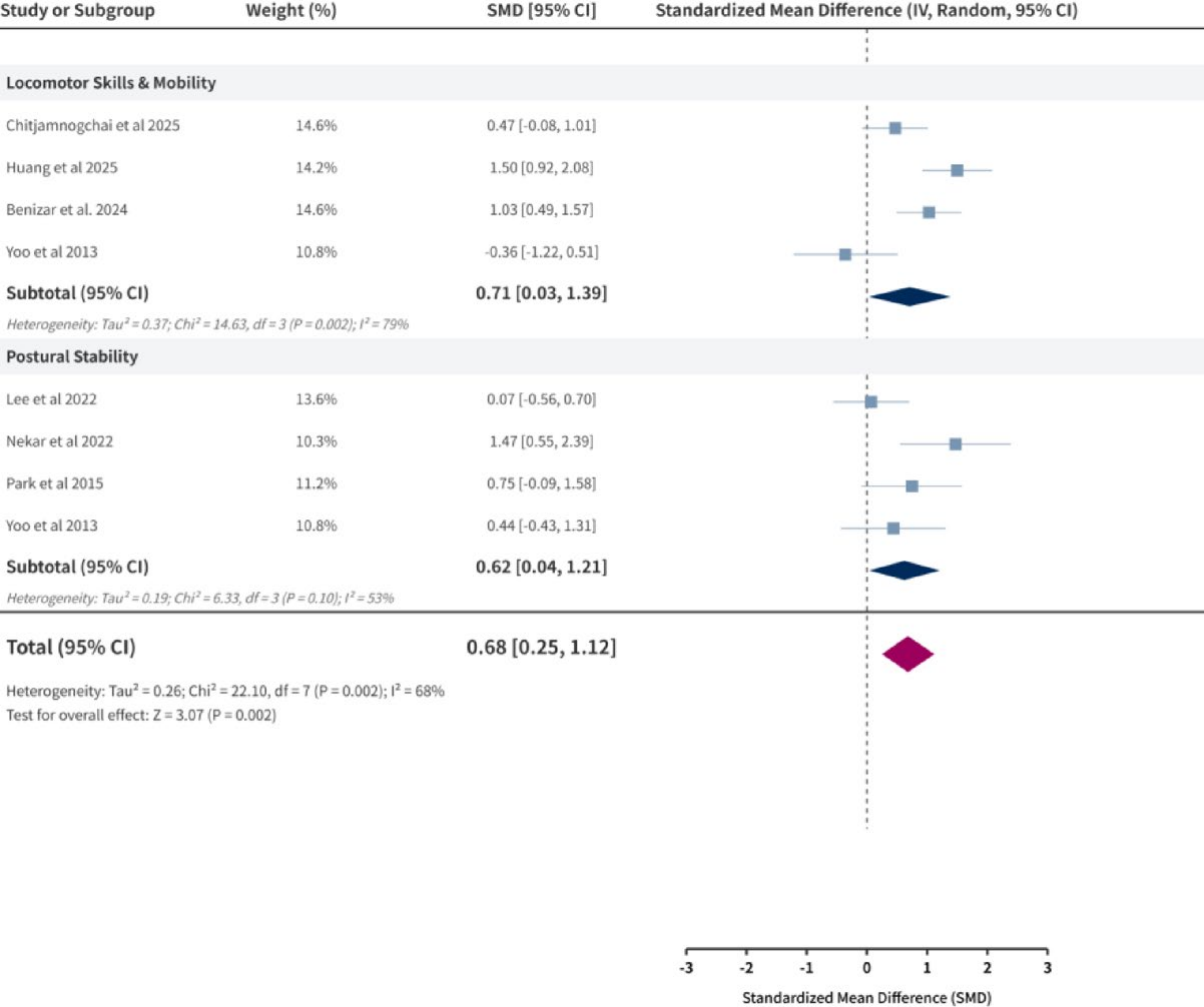
Stability & Functional Mobility meta.

Subgroup analyses in the stability and functional mobility domain showed significant improvements across both subcategories, with comparable effect magnitudes Fig. 7. The pooled SMD for locomotor skills and mobility was 0.71 (95% CI: 0.03, 1.39), and for postural stability was 0.62 (95% CI: 0.04, 1.21). The substantial overlap in confidence intervals between the two subgroups suggests comparable benefits of immersive AR/VR training for these abilities.Notably, substantial heterogeneity was observed within the locomotor skills and mobility subgroup (I^2^ = 79%), indicating considerable variability across studies. This may relate to differences in specific assessment tools or intervention protocols.

### Publication bias

**Fig. 8:**
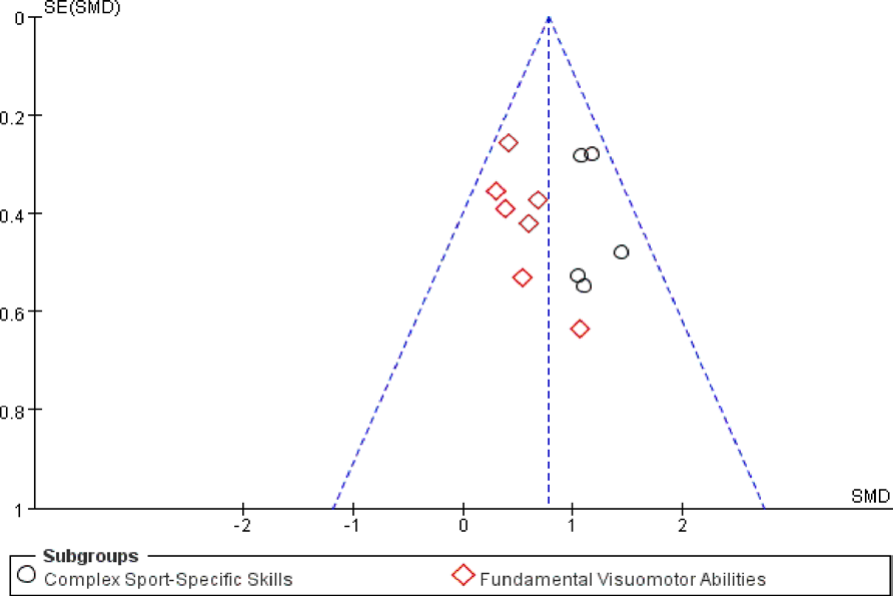
Funnel plot for publication bias in the object control and visuomotor skills domain.

**Fig. 9:**
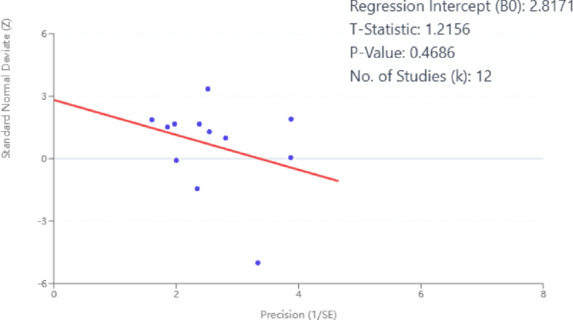
Egger’s regression test for asymmetry in the object control and visuomotor skills domain.

**Fig. 10:**
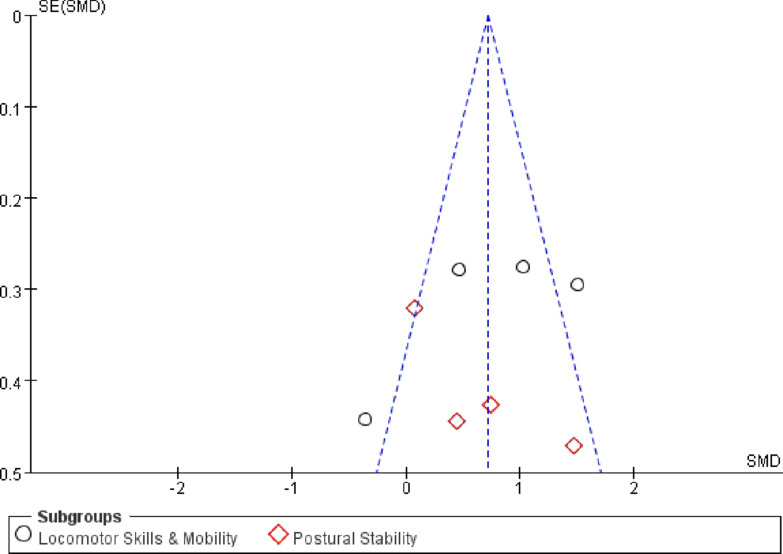
Funnel plot for publication bias in the stability and functional mobility domain.

Publication bias was assessed using funnel plots and Egger’s linear regression test for the included studies.For the object control and visuomotor skills domain Fig. 8, the funnel plot showed a largely symmetrical distribution of effect sizes around the pooled estimate, with no obvious asymmetry. Egger’s test confirmed this observation (t = 1.2156, *p* = 0.4686), indicating a low risk of publication bias Fig. 9.For the stability and functional mobility domain Fig. 10, the number of included studies was smaller (*n* = 8). Visual inspection of the funnel plot revealed a relatively symmetrical distribution, providing no clear evidence of publication bias.

## Discussion

This systematic review and meta-analysis evaluated the effects of immersive training delivered via AR and VR on motor competence. To enhance specificity, motor competence was stratified into two theoretically distinct constructs: object control and visuomotor skills (primarily open skills) and stability and functional mobility (primarily closed skills)^12,13^. Pooled analysis of 18 randomized and controlled intervention studies (*n* = 678 participants) demonstrated moderate to large positive effects in targeted domains compared with conventional training or no-intervention controls. A key contribution is the domain-specific synthesis, revealing that benefits vary substantially by task complexity: complex sport-specific skills showed the largest effects, while fundamental visuomotor tasks showed more modest improvements. These findings support immersive technologies as a powerful adjunct for motor learning, provided interventions are matched to the target domain^4,7,17,19^.

In the object control and visuomotor skills domain, subgroup analyses highlighted marked differences between complex sport-specific skills (SMD = 1.15, 95% CI 0.82–1.47; I^2^ = 0%) and fundamental visuomotor abilities (SMD = 0.39, 95% CI 0.10–0.67; I^2^ = 0%). Complex skills (e.g., ball control, table tennis swing, agility in dynamic settings) benefited more substantially. This aligns with ecological dynamics and perception-action coupling principles, where AR/VR provides dynamic, unpredictable constraints that closely simulate real-world demands^9,11,17,18,42^. Similar to motor-cognitive integration in dance training (Bläsing et al., 2018), high-fidelity simulations that preserve the full perception-action loop appear critical for transfer to real-world performance^18,43^. In contrast, fundamental visuomotor tasks in VR often isolate simple reaction time or hand-eye coordination from whole-body integration, reducing ecological validity and limiting transfer to complex athletic contexts^17,42^. Environmental fidelity and task representativeness emerge as key moderators of effectiveness in open-skill domains^39^.

For stability and functional mobility—predominantly closed skills performed in predictable, self-paced environments—immersive training yielded moderate and consistent benefits in locomotor skills and mobility (SMD = 0.71, 95% CI 0.03–1.39) and postural stability (SMD = 0.62, 95% CI 0.04–1.21). These effects are consistent with enhanced sensory feedback facilitating real-time postural and gait adjustments^4,8,44^. Substantial heterogeneity in the locomotor skills subgroup (I^2^ = 79%) likely reflects variability in intervention protocols, devices, dosages, outcome measures, and participant characteristics^45^. Consequently, although the pooled effect remained positive and robust in sensitivity analyses, clinicians and practitioners should exercise caution when generalizing these specific findings to broader populations or different settings until more standardized protocols are established^19,46,47^.

These positive effects must be interpreted in light of control conditions, which often produced comparable or statistically similar improvements^25,26^. This suggests that immersive AR/VR training is not necessarily superior to well-designed conventional approaches, but rather serves as a comparable or complementary adjunct. Its unique advantages — including overcoming space limitations, enhancing safety (e.g., fall risk reduction in older adults), enabling high-volume repetition of complex or high-risk scenarios, and increasing engagement through gamification — make it particularly valuable in resource-constrained or safety-critical contexts^7,48^. Thus, immersive training should be positioned as an enriched learning environment, capable of performing comparably to traditional methods in complex open-skill scenarios^18,39^.

Several limitations represent threats to validity rather than minor constraints. First, although domain-specific classification enhanced construct validity, mixed-skill protocols may introduce outcome construct mixing^25^, leading to classification bias. Second, small sample sizes (7–34) may inflate effect estimates and contribute to small-study effects. Third, substantial heterogeneity in some subgroups (e.g., I^2^^2^ = 79% in locomotor skills) reflects the nascent stage of AR/VR hardware, protocols, and outcome measures lacking standardization. Fourth, most studies examined short- to medium-term interventions (4–12 weeks), leaving long-term retention and real-world transfer underexplored^43,49^. Fifth, differences between passive (no-intervention) and active (conventional training) controls may exaggerate pooled effects; future analyses would benefit from stricter active-control designs to isolate immersive-specific effects. Sixth, potential non-independence of multiple outcomes within studies was not formally adjusted (e.g., multilevel modeling or correlation correction), and no formal certainty of evidence assessment (e.g., GRADE) was performed. Despite sensitivity analyses (including leave-one-out and exclusion of no-intervention controls) confirming robustness, these methodological factors limit broad generalizability^44^.

These limitations underscore the need for cautious interpretation and highlight priorities for future research: standardized protocols, long-term follow-up designs, rigorous active controls, advanced statistical handling of multiple outcomes and heterogeneity, and formal GRADE assessments.

In conclusion, immersive AR/VR training holds strong promise as a flexible adjunct in motor training and rehabilitation, particularly for enhancing complex open skills in resource-limited or high-risk settings and improving engagement across age groups^7^. Future high-quality RCTs should prioritize standardized outcomes, head-to-head AR vs. VR comparisons, long-term evaluations, neuroimaging to elucidate mechanisms, and formal evidence certainty assessments to optimize clinical and athletic application.

## Conclusion

This systematic review and meta-analysis demonstrates that immersive training primarily delivered through AR and VR is an effective approach for enhancing motor competence in healthy individuals and athletes, producing moderate to large positive effects across targeted domains (pooled SMD range: 0.62 to 1.15). However, benefits exhibit clear domain-specificity. In complex object control and visuomotor skills (open skills), high-fidelity immersive simulations imposing dynamic environmental constraints that closely replicate real-world motor demands yielded the largest gains (SMD = 1.15, 95% CI 0.82–1.47)^11,42^. In contrast, improvements in fundamental visuomotor abilities were more modest (SMD = 0.39, 95% CI 0.10–0.67), likely due to reduced ecological validity when isolating basic coordination from whole-body integration. For stability and functional mobility (closed skills), AR/VR interventions provided moderate and consistent benefits through enhanced multimodal sensory feedback supporting real-time corrections (locomotor skills/mobility SMD = 0.71, 95% CI 0.03–1.39; postural stability SMD = 0.62, 95% CI 0.04–1.21)^4,8^.

Critically, evidence indicates that AR/VR training performs comparably to well-designed conventional training rather than being uniformly superior. Sensitivity analyses confirmed the robustness of these findings, and publication bias was low. Immersive technologies should therefore not be viewed as a standalone replacement, but as a high-value, evidence-based adjunct capable of enriching and complementing traditional motor learning and rehabilitation environments, particularly in settings where conventional methods face practical constraints^1,7,18^.

In summary, AR and VR represent evidence-based, highly promising tools in sports science and rehabilitation. While moderate to substantial heterogeneity persists in some subgroups (e.g., I^2^ = 79% in locomotor skills), domain-specific analyses improve interpretability and practical relevance. To accelerate translation into routine practice, future research must move beyond basic efficacy demonstrations toward identifying for whom, under what conditions, and through which protocols immersive training is most effective. This requires standardized intervention guidelines, long-term follow-up to evaluate skill retention and real-world transfer, rigorous head-to-head randomized controlled trials comparing AR/VR with traditional methods, and formal certainty of evidence assessments (e.g., GRADE) to guide optimal integration into evidence-based motor training frameworks^17,19^.

## Supplementary Information

Below is the link to the electronic supplementary material.

Supplementary Material 1

## Data Availability

All data generated or analysed during this study are included in this published article and its supplementary information files.
